# DNA-free genome editing for *ZmPLA1* gene via targeting immature embryos in tropical maize

**DOI:** 10.1080/21645698.2023.2197303

**Published:** 2023-04-05

**Authors:** Sagar Krushnaji Rangari, Manjot Kaur Sudha, Harjot Kaur, Nidhi Uppal, Gagandeep Singh, Yogesh Vikal, Priti Sharma

**Affiliations:** a School of Agricultural Biotechnology, Punjab Agricultural University, Ludhiana, Punjab, India; b Department of Plant Breeding and Genetics, Punjab Agricultural University, Ludhiana, Punjab, India

**Keywords:** Crispr/Cas9, haploid induction, immature embryos, maize, Ribonucleoprotein (RNP) complex

## Abstract

Doubled haploid (DH) production accelerates the development of homozygous lines in a single generation. In maize, haploids are widely produced by the use of haploid inducer Stock 6, earlier reported in 1959. Three independent studies reported haploid induction in maize which is triggered due to a 4 bp frame-shift mutation in *matrilineal* (*ZmPLA1*) gene. The present study was focused on the generation of mutants for *ZmPLA1* gene in maize inbred line LM13 through site-directed mutagenesis via CRISPR/Cas9-mediated ribonucleoprotein (RNP) complex method to increase the haploid induction rate. Three single guide RNAs (sgRNAs) for the *ZmPLA1* gene locus were used for transforming the 14 days old immature embryos via bombardment. 373 regenerated plants were subjected to mutation detection followed by Sanger’s sequencing. Out of three putative mutants identified, one mutant depicted one base pair substitution and one base pair deletion at the target site.

In light of the changing climatic scenario, it is important to accelerate climate-resilient line development. Doubled haploid (DH) technology based on *in vivo* haploid induction and the successive doubling of haploids is a promising method to develop homozygous lines in 2–3 generations.^1^^,^^2,3^ Thus the DHs can acquire conventional plant breeding to speed up the breeding of high yielding varieties and resistant to the biotic and abiotic stresses.

The successful *in vivo* maternal haploid induction is triggered by using the inducer line as male, which is a unique mechanism in maize DH production system. CRISPR-Cas9 system has been established as a revolutionary technique with wide applications in plant biology.^4^ In maize, genome modification has been reported previously for different traits.^5^ Many quantitative trait loci (QTLs) which significantly affect the haploid induction rate (HIR) have been mapped.^6^ Among these *qhir1* QTL locus has the most significant effect on the haploid induction.

Prigge *et al*.^7^ reported that two key quantitative trait loci, *qhir1* and *qhir8*, lead to high-frequency haploid induction in maize. Fine mapping and sequence analysis studies of these QTLs led to the conclusion that mutations in PHOSPHOLIPASE A1 (*ZmPLA1*)^8^ and DOMAIN OF UNKNOWN FUNCTION 679 MEMBRANE PROTEIN (*ZmDMP*)^9^ have been shown to generate haploids in maize. Both *ZmDMP* and *ZmPLA1* have shown similar expression patterns and subcellular localization in maize. Recent findings reveal that knockout of *ZmDMP* along with *ZmPLA1* triggered haploid induction and exhibited a greater ability to increase the HIR by 5–6 folds.^10^

There are some newly developed protocols which can be used for the regeneration of the plantlets from the immature embryos of maize,^11^ *In vitro* transcription of the sgRNA,^12^ the modified gene gun bombardment technique for an effortless gene transformation^13^ and *in vitro* cleavage of the DNA using CRISPR/Cas9 RNP complex for the pre-validation, functionality and efficiency of CRISPR genome editing system.^14^ The present study aims at generating mutants for *ZmPLA1* gene in maize inbred line of PAU (LM13) through RNP complex-based genome editing to obtain higher HIR.

## Materials and Methods

Maize inbred line LM13 (JCY-3-7-1-1-1) was selected to generate the mutants for the target gene, as this line is better responsive to the agronomical practices and a good combiner in hybrid breeding programme. *ZmPLA1* gene from the LM13 was cloned, sequenced and aligned to *ZmPLA1* locus (GRMZM2G471240) and conserved regions were identified to design sgRNA. The guide RNA was designed using CRISPR Plant v2.0 software (http://crispr.hzau.edu.cn/CRISPR2/) and comparative analysis was done with other software known as CHOP CHOP (http://chopchop.cbu.uib.no/) (Table 1). sgRNA synthesis was followed according to the protocols provided with Thermo Fisher Kit. T7 promotor region was used for the *in vitro* transcription of sgRNA from its DNA template. *in vitro* transcribed product purified and maintained for further experiments. Ribonucleoprotein complex was prepared by taking 2 µg of Truecut Cas9 protein (Thermofisher) and 2 µg of sgRNA at optimal 1:1 concentration and was used for the *in vitro* cleavage assay and then for the bombardment reactions. *in vitro* cleavage assay was performed, to check the cleavage efficiency for all these sgRNAs.

**Table 1.** List of sgRNA’s (with protospacer adjacent motifs) with their parameters.

S. No.	sgRNAs+ PAM sequence	GC content (%)	Off-targets score	Mismatch	On-score	Strand	Exon
1.	5”GACTACTTCGACTACATCGCCGG3”	50	99	≈0–4	0.60	Positive	1
2.	5”GAGCATCCTCGGCGAGACGAGGG3”	65	101	≈3–4	0.57	Positive	2
3.	5”GTTGCAGGAGCTGGACGGACCGG3”	70	317	≈3–4	0.09	Positive	1

Immature embryos were placed on the 0.4 M mannitol containing osmoticum MS medium and used for bombardments. These bombarded embryos were then transferred into the half MS medium to obtain the desired growth of plantlets. Genomic DNA was isolated from the regenerated plantlets using CTAB method.^15^ The *ZmPLA1* gene-specific primers were designed for first exon using Primer 3 software (*ZmPLA*EX1MDF–5“GTCCATGCAATACCTGTAGC3,”

*ZmPLA*EX1MDR–5“AAAGTGGTTGATGTCCTTGG3,”

*ZmPLA*EX1MDF1–5“CCATGCAATACCTGTAGCACG3,”

*ZmPLA*EX1MDR1–5“GGATGGATGCAAGAACAATGG3”). PCR amplified products from all the 288 regenerated plantlets were resolved on 2.5% agarose gel. Cleavage mutation detection assay was performed by GeneArt® genomic cleavage detection kit (ThermoFisher Scientific) to identify the putative mutants. The cleavage detection enzyme T7E1 recognizes and cleaves the heteroduplex loops formed by insertion or deletion of nucleotides. Due to limitations of this assay rest of the remaining samples were sent for the Sanger’s sequencing in duplicates. After sequencing, various bioinformatic tools like Chromas software, clustalX 2.0 software, BLAST analysis by NCBI, CRISPR ID and DsDecodedM, were used to align the sequences with reference control (LM13) to identify any mutations like insertion/deletion.The monoallelic and biallelic mutations were detected using the DsDecodedM online web tool. ExPASy and ORF finder were used to track the disturbance in open reading frame or the replacements and rearrangements in amino acids.

## Results and Discussion

In the present study, efforts were made to edit the *ZmPLA1* locus through site-directed mutagenesis to generate a novel haploid inducer stock in tropical maize background. As previously reported, haploids were produced using crosses to Stock 6^16^ and the mechanism of haploid induction was studied later on by three independent laboratories.^8,17,18^ The mechanism of haploid induction is triggered by a 4 bp mutation in the *ZmPLA1* gene^8,18^ and further edits in this gene could lead to increase in haploid induction rate of 6–7%.

**Figure 1.**
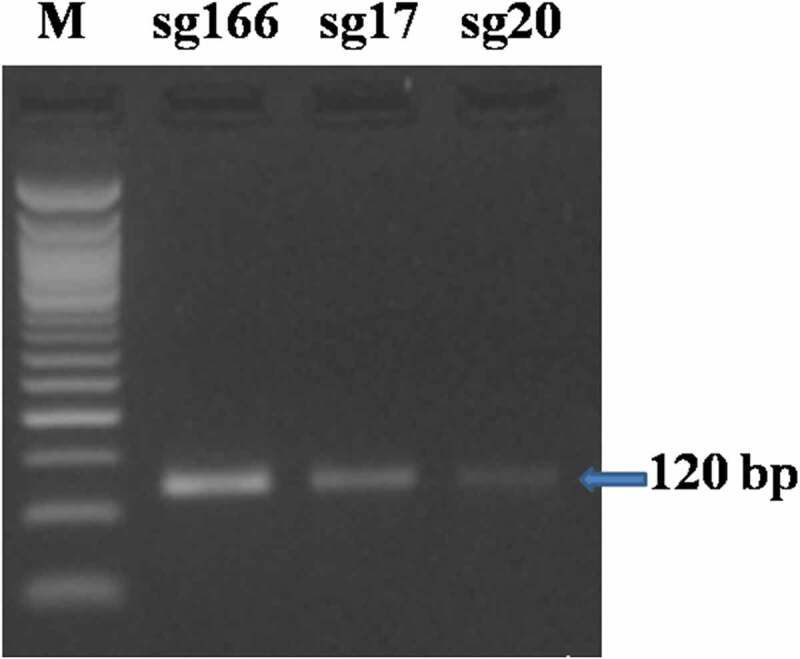
sgRNA DNA template−120 bp fragment (*M* = 50 bp ladder).

**Figure 2.**
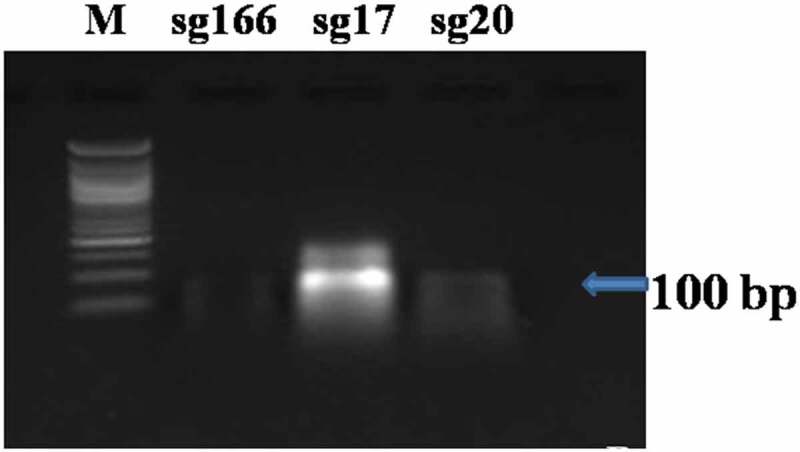
*in vitro* transcription of sgRNA- 100 bp fragment (*M* = 50 bp ladder).

The sgRNA DNA templates of 120 bp for the sgRNAs namely sg166, sg17 and sg20 were synthesized followed by *in vitro* transcription that resulted in 100 bp intact CRISPR fragment of sgRNAs (Figs. 1 and 2). Similarly, Hu *et al*.^19^ observed 120 bp of sgRNA DNA template synthesized using four overlapping primers for targeting the EGFR genes. Moreover, Liang *et al*.^20^ transcribed a 100 bp intact fragment of sgRNA from the template while working on isolated cell lines of Jurkat cells. Jinek *et al*.^21^ observed cleaved fragments of ≈ 2230 bp and≈3100 bp from 5332 bp intact fragment of human clathrin light chain (*CLTA*) gene which was an indicator of Cas9 mediated cleavage. Therefore, *in vitro* cleavage assay was performed on the transcribed gRNAs for determining their cleavage efficiency. The assay resulted in two cleaved bands of size≈210 bp and≈165 bp and≈230 and≈150 bp in case of sg166 and sg17 respectively. However, the sgRNA sg20 resulted in an intact band of 376 bp depicting no proper cleavage (Supp Fig. 3); hence the sgRNA sg166 and sg17 were selected for further study.

**Figure 3.**
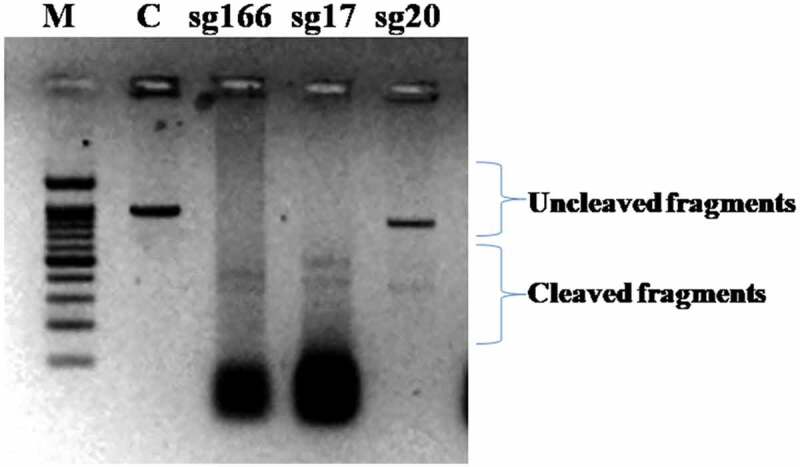
*in vitro* cleavage assay gel picture.

**(M = 50 bp ladder, C= uncleaved control)**

Previous reports available in maize suggest successful regeneration from mature embryos,^22^ nodal culture,^23^ splited seeds,^24^ and immature embryos as an explant.^25–27^ However, immature embryos are the material of choice in maize for the efficient production of transgenic lines through particle bombardment or *Agrobacterium* transformation.^28^ The advantages of particle bombardment over *Agrobacterium* mediated transformation were studied by Mookkan.^13^ In the present study, a total of 2315 embryos were bombarded using both the sgRNAs and 373 plants were regenerated and survived upto hardening. Malini *et al*.^29^ reported that the concentrations of BAP (0.5, 1.0 and 1.5 mg/l), NAA (0.1, 0.2 and 0.3 mg/l) and kinetin (1.0 mg/l) to be used for plant regeneration in case of immature embryos. Whereas Guruprasad *et al*.^28^ regenerated plantlets from immature embryos using MS medium formulations.

The cleavage detection assay was performed on the few samples to detect the mutations in the targeted region along with LM13 (Non-transformed) as a control. The cleaved bands in the positive control validated the efficiency of the sgRNAs; meanwhile, the single band in negative control LM13 against the two cleaved bands in the sample no. 102 showed the presence of mutation. All the edited plants (288) obtained were further validated through Sanger’s Sequencing. The sequences obtained were aligned with the LM13 sequence (as reference) using ClustalX 2.0. Mutations were identified in only three samples after alignment of the sequences. Two sequences were found to have mutations just near the target site of sgRNA and only one sequence showed one nucleotide of substitution and deletion at the target site of sgRNA. The results of the BLAST for the edited plant no. 258, 287 and 21 are given in Supp Fig 4, Figs. 5 , and 6. In case of a diploid organism, it is very important to disrupt both the copies of a gene to suppress its expression. Homozygous biallelic mutant for the plant no. 21 has the substitution in both the alleles as depicted in Supp Fig. 7. The monoallelic mutant in the plant no. 258 has one base substitution in a single allele as represented in Fig. 8.

**Figure 4.**
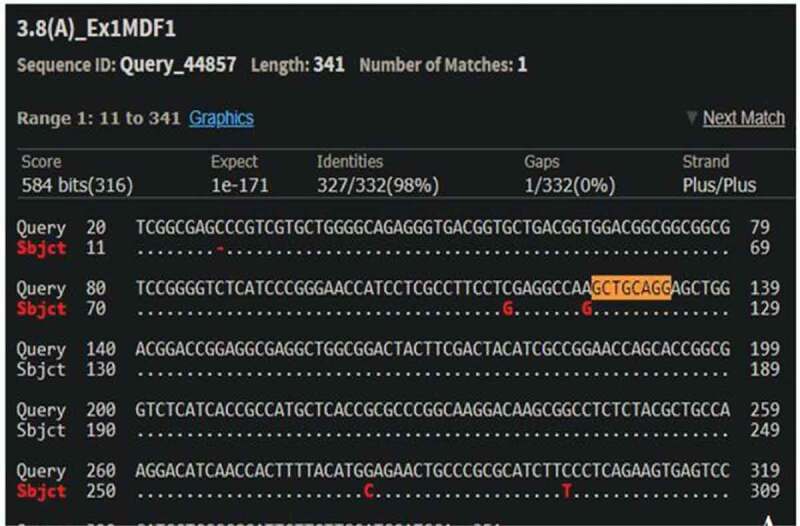
BLAST result of plant no. 258 with parent LM13 (NCBI BLAST).

**One base pair substitution at just near the target sequence**

**Figure 5.**
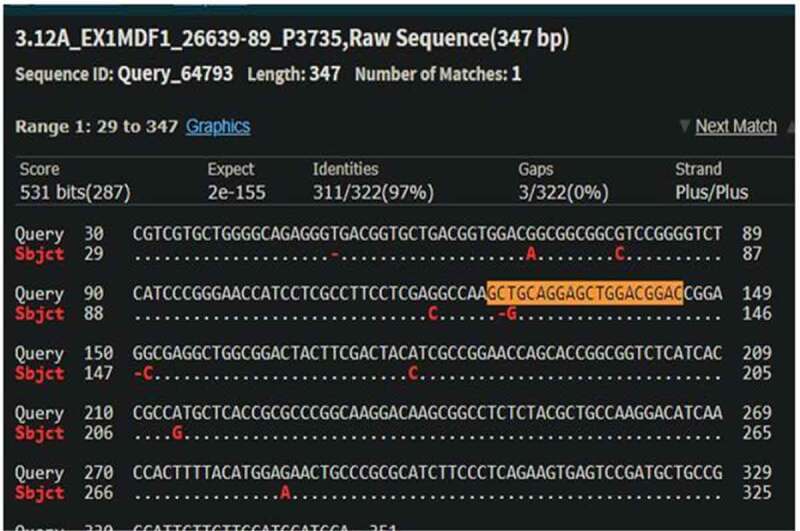
BLAST result of plant no. 287 with parent LM13 (NCBI BLAST).

**One base pair of deletion and one base pair of substitution at the target region**

**Figure 6.**
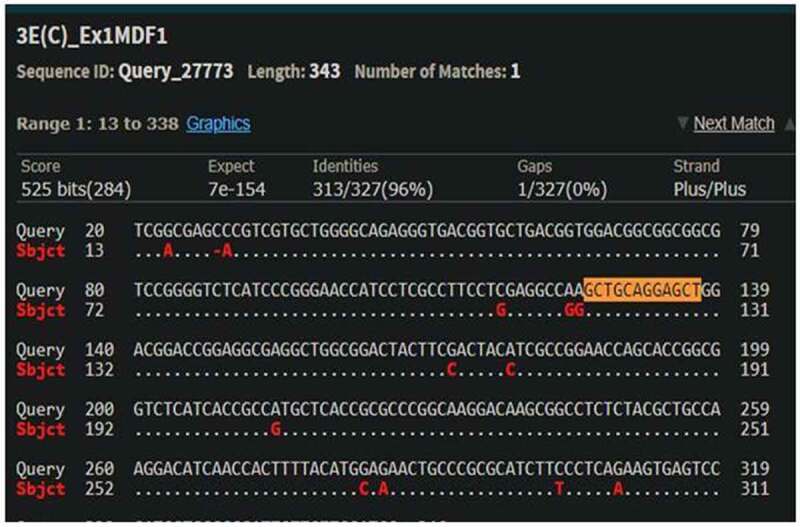
BLAST result of plant no. 21 with parent LM13 (NCBI BLAST).

**Two base pair substitution at just near the target region**

**Figure 7.**
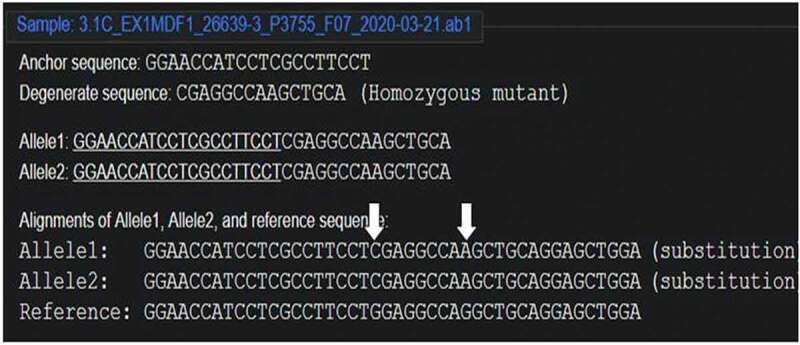
Homozygous biallelic mutant for plant no. 21 (DsDecodedm online web tool).

**Figure 8.**
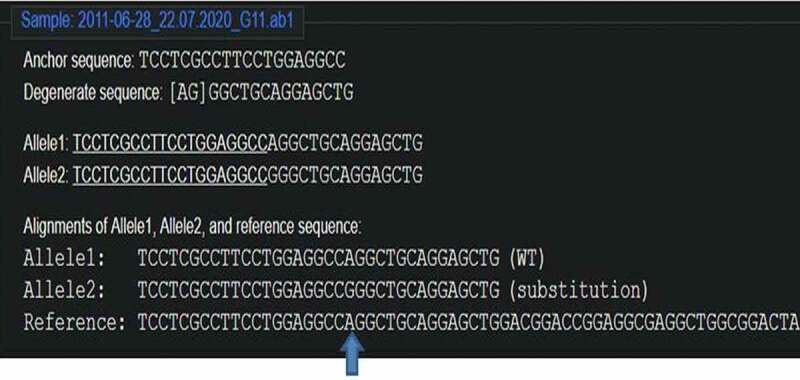
the monoallelic mutant in the plant no. 258 (DsDecodedm online web tool).

At the protein level, the ExPASy tool had shown the disruption of the 33 amino acids chain in the putative mutant sample no. 287. These results were cross-checked with the ORF finder online web tool. The putative sample was then self-pollinated to obtain T_1_ seeds from the edited T_0_ mutant. A single cob containing 48 seeds was obtained from this mutated plant. These T_1_ edited seeds are being maintained for the generation of T_2_ progeny and further needs screening to evaluate the haploid induction ability by crossing with different maize inbred lines.

Although the undesirable effects coupled with backcrossing and other conventional methods are reduced using biolistic delivery of RNP complexes into plant cells. Current methods of genetic transformation together with low efficiency of DNA delivery into target regions and less regeneration rate of plants create hindrance for editing plant genomes. Feng *et al*.^30^ targeted a marker gene *Zmzb7* for genome editing which is responsible for leaf coloration in plants. Wang *et al*.^31^ edited the *ZmLG1* gene, which is a major determinant of leaf angle in maize. The loss of function of this gene resulted in a reduction of leaf angle due to the absence of auricle and ligules. Our research shows the successful utilization of *ZmPLA1* gene for inducing haploid induction in tropical maize line. There are recent studies based on the identification of genes such as *ZmDMP*^9^ and *ZmPLD3*^10^ that are responsible for haploid induction.

*In-vitro* cleavage assay can be successfully used to pre-evaluate the efficiency of the designed sgRNA’s in the CRISPR Cas9 genome editing experiments. Immature embryos about 12–14 days after pollination can be successfully used for the gene gun bombardment via ribonucleoprotein complex delivery. Disruption of one of the 33 AA protein frame in first exon shows possibility of haploid induction.

## Supplementary Material

Supplemental Material

## Abbreviations

T1: First Transgenic generation
LM13: Ludhiana maize 13
bp: Base pair
CRISPR: Clustered Regularly Interspaced Short Palindromic Repeats
Cas9: CRISPR-associated protein 9

## References

[1] Forster BP, Thomas W. Doubled haploids in genetics and plant breeding. Plant Breed Rev. 2005;25:57–88.

[2] Chang M, Coe E. Molecular genetics approaches to maize improvement. In: L KA A LB, editors. Biotechnology in agriculture and forestry. Berlin, Heidelberg, Germany: Springer; 2009. pp. 127–42.

[3] Gordillo G, Geiger H. Alternative recurrent selection strategies using doubled haploid lines in hybrid maize breeding. Crop Sci. 2008;48(3):911–22. doi:10.2135/cropsci2007.04.0223.

[4] Shan Q, Zhang Y, Chen K, Zhang K, Gao C. Creation of fragrant rice by targeted knockout of the OsBADH2 gene using TALEN technology. Plant Biotechno. 2015;**l**13(6):791–800. doi:10.1111/pbi.12312. 25599829

[5] Svitashev S, Schwartz C, Lenderts B, Young JK, Cigan MA. Genome editing in maize directed by CRISPR–Cas9 ribonucleoprotein complexes. Nat Com. 2016;7(1):1–7. doi:10.1038/ncomms13274. PMC5116081 27848933

[6] Hu H, Schrag TA, Peis R, Unterseer S, Schipprack W, Chen S, Lai J, Yan J, Prasanna M, Nair SK, et al. The genetic basis of haploid induction in maize identified with a novel Genome-Wide association method. Genetics. 2016;202(4):1267–76. doi:10.1534/genetics.115.184234. 26896330 PMC4905542

[7] Prigge V, Xu X, Li L, Babu R, Chen S, Atlin GN, Melchinger AE. New insights into the genetics of in vivo Induction of maternal haploids, the backbone of doubled haploid technology in maize. Genetics. 2012;190:781–93. doi:10.1534/genetics.111.133066. 22135357 PMC3276611

[8] Kelliher T, Starr D, Richbourg L, Chintamanani S, Delzer B, Nuccio ML, Green J, Chen Z, McCuiston J, Wang W, et al. Matrilineal, a sperm-specific phospholipase, triggers maize haploid induction. Nature. 2017;542(7639):105–09. doi:10.1038/nature20827. 28114299

[9] Zhong Z, Liu C, Xi Q, Jiao Y, Wang D, Wang Y, Liu Z, Chen C, Chen B, Tian X, et al. Mutation of ZmDMP enhances haploid induction in maize. Nature Plant. 2019;5(6):575–80. doi:10.1038/s41477-019-0443-7. 31182848

[10] Li Y, Lin Z, Yue Y, Zhao H, Fei X, Liu C, Chen S, Lai J, Song W, Song W. Loss-of-function alleles of ZmPLD3 cause haploid induction in maize. Nature Plant. 2021;7(12):1579–88. doi:10.1038/s41477-021-01037-2. PMC8677622 34887519

[11] Abhishek A, Chikkappa GK, Ravindra N, Meenakshi B, Ramteke PW, Pradyumn K, Sain D, Sai KR. Differential effect of immature embryo’s age and genotypes on embryogenic type II callus production and whole plant regeneration in tropical maize inbred lines (*Zea mays* l.). Ind J Gen Plant Breed. 2014;74(3):317–24. doi:10.5958/0975-6906.2014.00849.9.

[12] Beckert B, Masquida B. Synthesis of RNA by *in vitro* transcription. Method Mol Bio. 2011;703:29–41. 10.1007/978-1-59745-248-9_3 21125481

[13] Mookkan M, Mookkan. Particle bombardment – mediated gene transfer and GFP transient expression in Setaria viridis. Plant Signalling Behav. 2018;13(4):67–76. doi:10.1080/15592324.2018.1441657. PMC5933905 29621423

[14] Mehravar M, Shirazi A, Mehrazar MM, Nazari M. *In vitro* pre-validation of gene editing by CRISPR/Cas9 ribonucleoprotein. Avicenna J Med Biotechnol. 2018;11:259–63. PMC6626505 31380000

[15] Murray MG, Thompson WF. Rapid isolation of high molecular weight plant DNA. Nucleic Acids Res. 1980;8(19):4321–25. doi:10.1093/nar/8.19.4321. 7433111 PMC324241

[16] Coe E. A line of maize with high haploid frequency. Amer Naturalist. 1959;93(873):381–82. doi:10.1086/282098.

[17] Gilles LM, Khaled A, Laffaire J, Chaignon S, Gendrot G, Laplaige J, Berges H, Beydon G, Bayle V, Barret P, et al. Loss of pollen-specific phospholipase *not like dad (NLD)* triggers gynogenesis in maize. Embo J. 2017;36(6):1–11. doi:10.15252/embj.201796603. 28228439 PMC5350562

[18] Liu H, Ding Y, Zhou Y, Jin W, Xie K, Chen L. CRISPR-P 2.0: an improved CRISPR-Cas9 tool for genome editing in plants. Mol Plant. 2017;10(3):530–32. doi:10.1016/j.molp.2017.01.003. 28089950

[19] Hu Z, Wang L, Shi Z, Jiang J, Li X, Chen Y, Li K, Luo D. Customized one-step preparation of sgRNA transcription templates via overlapping PCR Using short primers and its application in vitro and in vivo gene editing. Cell Bisci. 2019;9(1):1–7. doi:10.1186/s13578-019-0350-7. PMC6814055 31673328

[20] Liang X, Potter J, Kumar S, Zou Y, Quintanilla R, Sridharan M, Carte J, Chen W, Roark N, Ranganathan S, et al. Rapid and highly efficient mammalian cell engineering via Cas9 protein transfection. J Biotechnol. 2015;7098:1–10. doi:10.1016/j.jbiotec.2015.04.024. 26003884

[21] Jinek M, Chylinski K, Fonfara I, Hauer M, Doudna JA, Charpentier E. A programmable dual-RNA–Guided DNA endonuclease in adaptive bacterial immunity. Science. 2012;337(6096):816–21. doi:10.1126/science.1225829. 22745249 PMC6286148

[22] Huang X, Wei Z. High-frequency plant regeneration through callus initiation from mature embryos of maize (*Zea Mays* L.). Plant Cell Rep. 2004;22(11):793–800. doi:10.1007/s00299-003-0748-9. 15022014

[23] Vladimir S, Gilbertson L, Adae P, Duncan D. Agrobacterium-mediated transformation of seedling-derived maize callus. Plant Cell Rep. 2006;25(4):320–28. doi:10.1007/s00299-005-0058-5. 16252091

[24] Al-Abed D, Rudrabhatla S, Talla R, Goldman S. Split seed: a new tool for maize researchers. Planta. 2006;223(6):1355–60. doi:10.1007/s00425-006-0237-9. 16489455

[25] Bohorova NE, Luna B, Brito RM, Huerta LD, Hoisington DA. Regeneration potential of tropical, subtropical, mid-altitude and highland maize inbreds. Maydica. 1995;4:275–81.

[26] Duncan DR, Williams ME, Zehr BE, Widholm JM. The production of callus capable of plant regeneration from immature embryos of numerous *Zea mays* genotypes. Planta. 1985;165:322–32. doi:10.1007/BF00392228. 24241136

[27] Furini A, Jewell DC. Somatic embryogenesis and plant regeneration from immature and mature embryos of tropical and subtropical *Zea Mays* L. Genotypes. Maydica. 1994;39L:155–64.

[28] Guruprasad M, Sridevi V, Kumar BK, Kumar G, Kumar S. An efficient regeneration and genetic transformation of maize through Agrobacterium and particle bombardment in immature embryos. Indian J Agric Res. 2016;50:414–20.

[29] Malini N, Kumar S, Ramakrishnan SH. Regeneration of Indian maize genotypes (*Zea mays* L.) from immature embryo culture through callus induction. J Appl Nat Sci. 2015;7(1):131–37. doi:10.31018/jans.v7i1.576.

[30] Feng C, Yuan J, Wang R, Liu Y, Birchler JA, Han F. Efficient targeted genome modification in maize using the CRISPR/Cas9 system. J Genet Genomics. 2015;43(1):37–43. doi:10.1016/j.jgg.2015.10.002. 26842992

[31] Wang J, Meng X, Hu X, Sun T, Li J, Wang K, Yu H. xCas9 expands the scope of genome editing with reduced efficiency in rice. Plant Biotechnol J. 2019;17(4):709–11. doi:10.1111/pbi.13053. 30549238 PMC6419569

