# Supplementary material for: DNA-free genome editing for ZmPLA1 gene via targeting immature embryos in tropical maize
Source: GM Crops Food. 2023 Apr 5;14(1):1–7. doi: 10.1080/21645698.2023.2197303 (PMC10761150; doi:10.1080/21645698.2023.2197303)
Supplement: Supplemental Material [file KGMC_A_2197303_SM2019.docx]

| 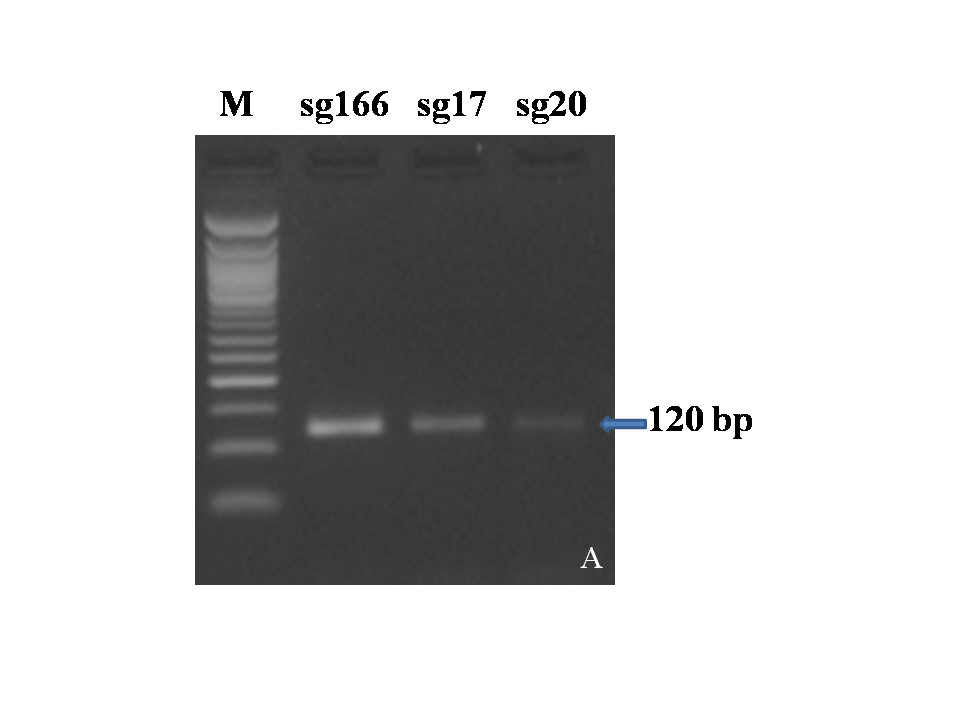 |
| --- |
| **Fig1.TIF sgRNA DNA template -120 bp fragment (M= 50 bp ladder)** |

| 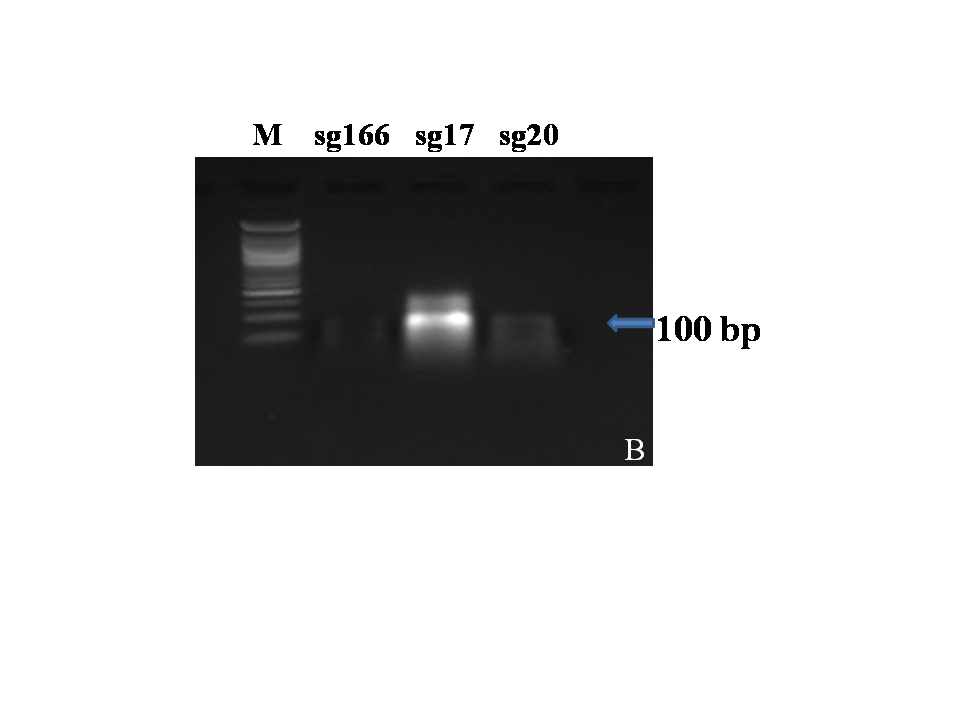 |
| --- |
| **Fig2.TIF *in vitro* transcription of sgRNA’s- 100 bp fragment (M= 50 bp ladder)** |

| 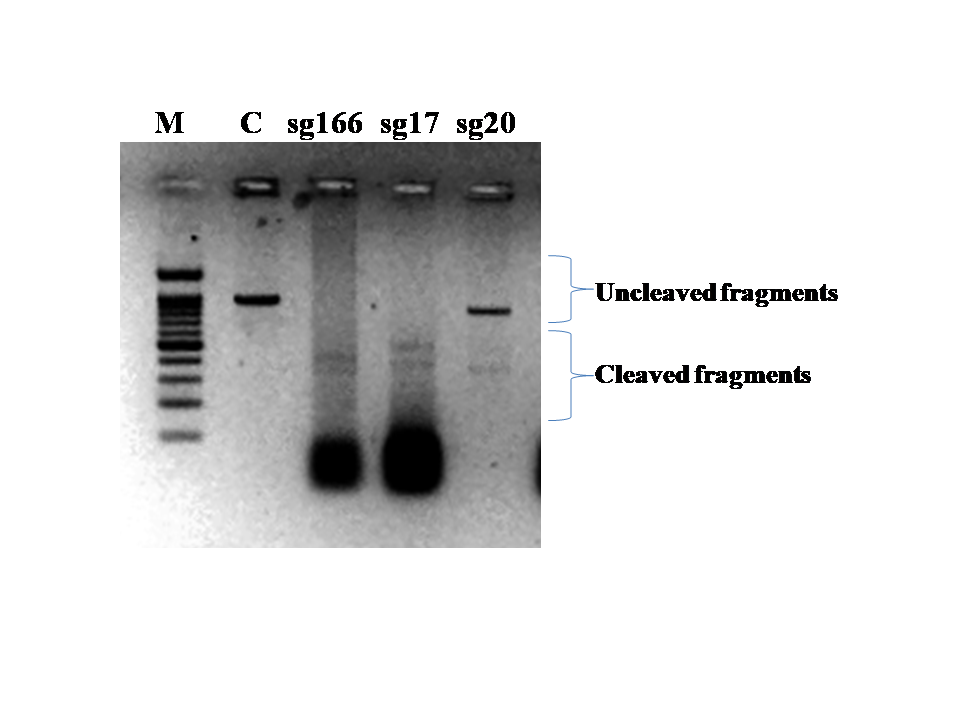 |
| --- |
| **Fig3*.*TIF *in vitro* cleavage assay gel picture**  **(M= 50 bp ladder, C= uncleaved control)** |
| 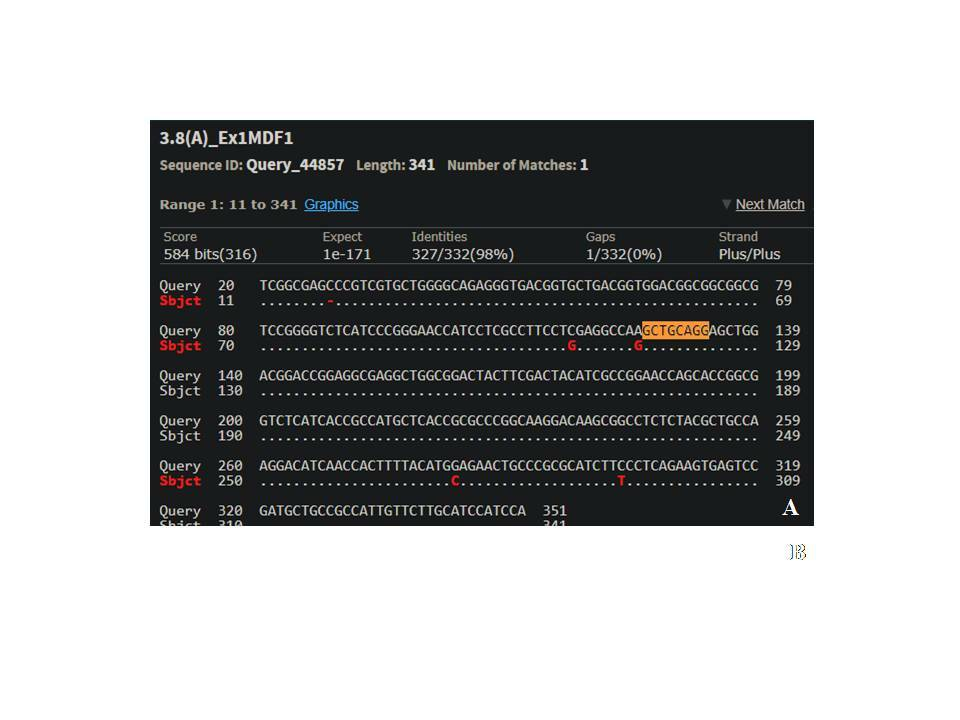 |
| **Fig4.TIF BLAST result of plant no. 258 with parent LM13 (NCBI BLAST)**  **One base pair substitution at just near the target sequence** |

| 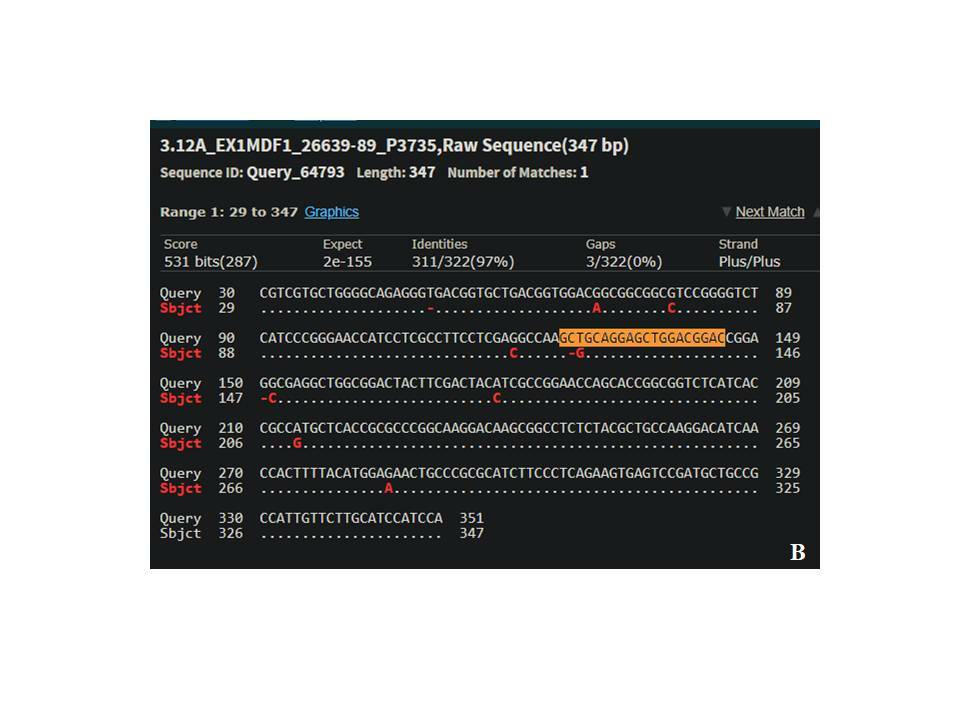 |
| --- |
| **Fig5.TIF BLAST result of plant no. 287 with parent LM13 (NCBI BLAST)**  **One base pair of deletion and one base pair of substitution at the target region** |

| 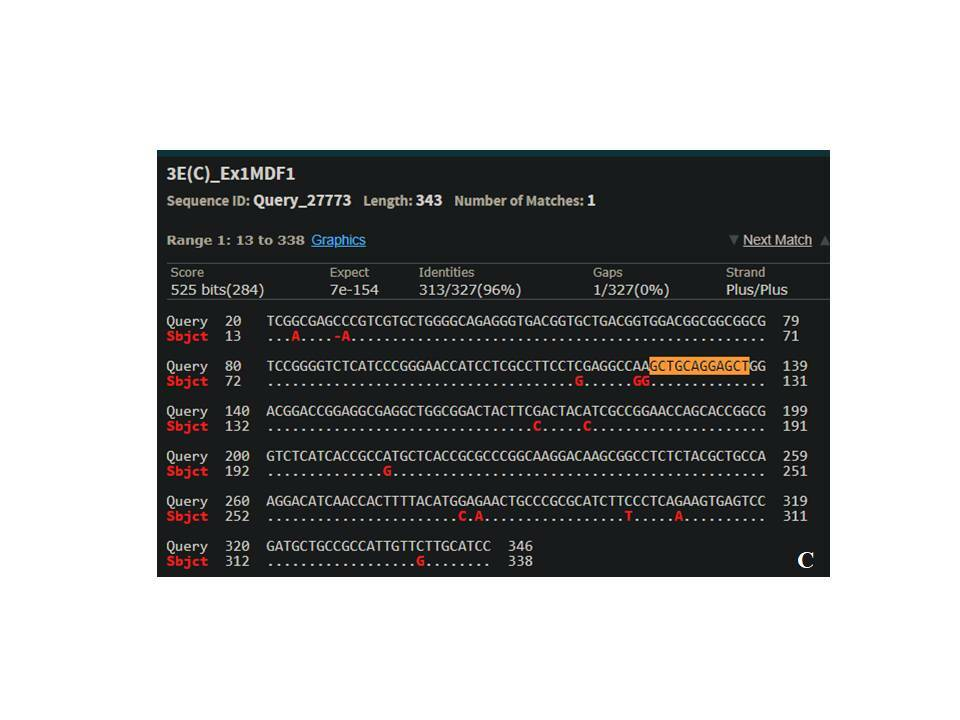 |
| --- |
| **Fig6.TIF BLAST result of plant no. 21 with parent LM13 (NCBI BLAST)**  **Two base pair substitution at just near the target region** |
| 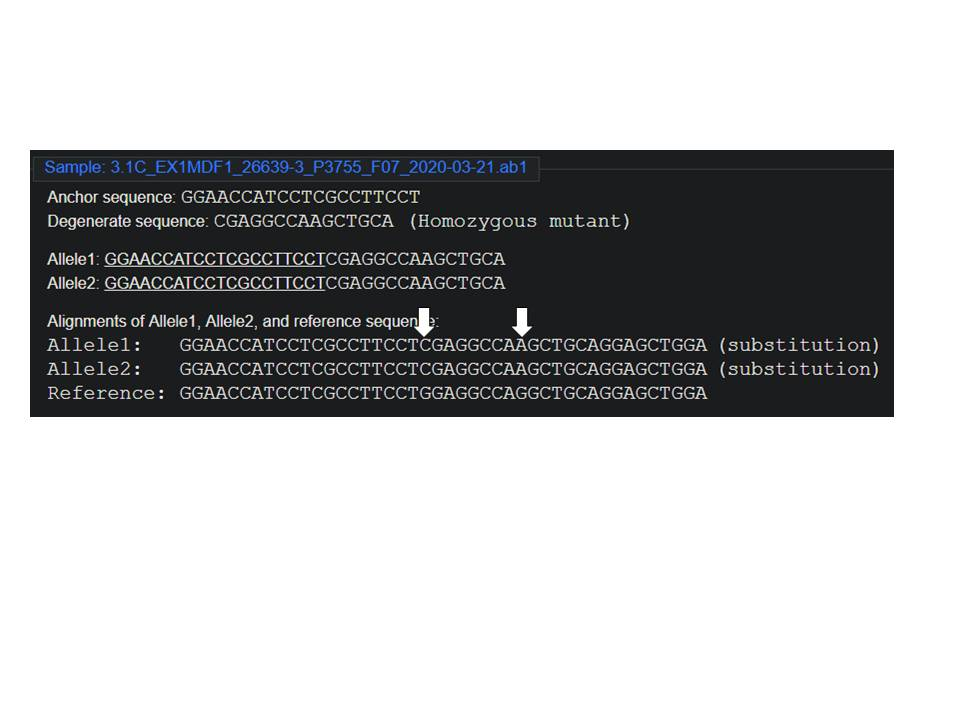 |
| **Fig7.TIF Homozygous biallelic mutant for plant no. 21 (DsDecodedM online web tool)** |

| 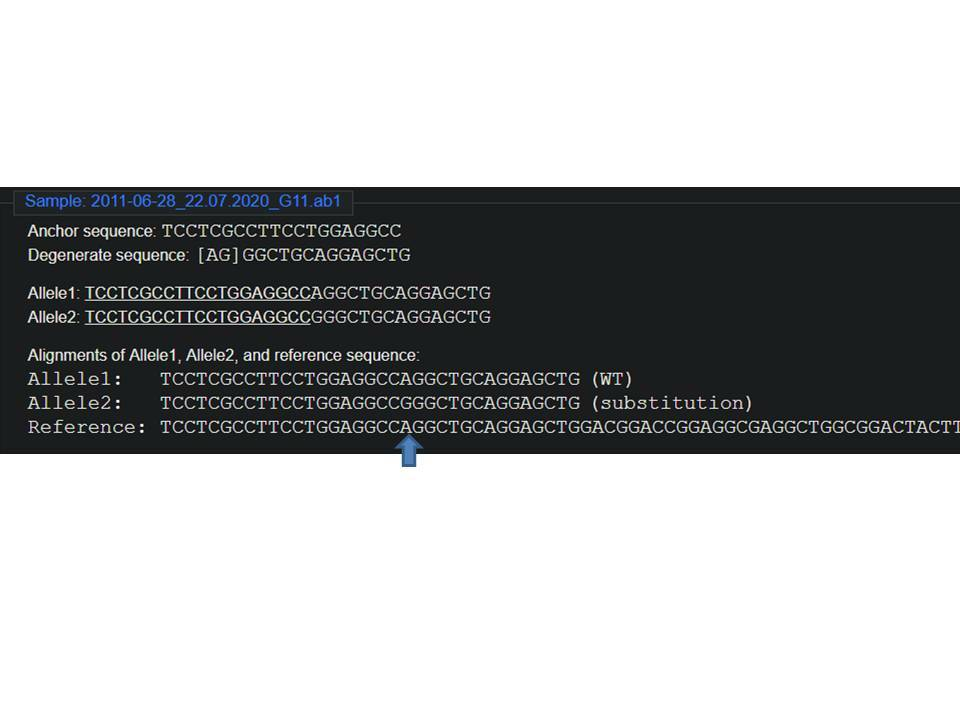 |
| --- |
| **Fig8.TIF The monoallelic mutant in the plant no. 258 (DsDecodedM online web tool)** |
